# A deep learning model incorporating spatial and temporal information successfully detects visual field worsening using a consensus based approach

**DOI:** 10.1038/s41598-023-28003-6

**Published:** 2023-01-19

**Authors:** Jasdeep Sabharwal, Kaihua Hou, Patrick Herbert, Chris Bradley, Chris A. Johnson, Michael Wall, Pradeep Y. Ramulu, Mathias Unberath, Jithin Yohannan

**Affiliations:** 1 Wilmer Eye Institute, Johns Hopkins University School of Medicine, Baltimore, MD USA; 2 Malone Center for Engineering, Johns Hopkins University, Baltimore, MD USA; 3 Department of Ophthalmology and Visual Sciences, University of Iowa, Iowa City, IA USA

**Keywords:** Predictive markers, Glaucoma, Translational research, Machine learning

## Abstract

Glaucoma is a leading cause of irreversible blindness, and its worsening is most often monitored with visual field (VF) testing. Deep learning models (DLM) may help identify VF worsening consistently and reproducibly. In this study, we developed and investigated the performance of a DLM on a large population of glaucoma patients. We included 5099 patients (8705 eyes) seen at one institute from June 1990 to June 2020 that had VF testing as well as clinician assessment of VF worsening. Since there is no gold standard to identify VF worsening, we used a consensus of six commonly used algorithmic methods which include global regressions as well as point-wise change in the VFs. We used the consensus decision as a reference standard to train/test the DLM and evaluate clinician performance. 80%, 10%, and 10% of patients were included in training, validation, and test sets, respectively. Of the 873 eyes in the test set, 309 [60.6%] were from females and the median age was 62.4; (IQR 54.8–68.9). The DLM achieved an AUC of 0.94 (95% CI 0.93–0.99). Even after removing the 6 most recent VFs, providing fewer data points to the model, the DLM successfully identified worsening with an AUC of 0.78 (95% CI 0.72–0.84). Clinician assessment of worsening (based on documentation from the health record at the time of the final VF in each eye) had an AUC of 0.64 (95% CI 0.63–0.66). Both the DLM and clinician performed worse when the initial disease was more severe. This data shows that a DLM trained on a consensus of methods to define worsening successfully identified VF worsening and could help guide clinicians during routine clinical care.

## Introduction

Glaucoma is the leading cause of irreversible blindness worldwide and early identification of worsening is critical for prevention^1,2^. Visual field (VF) testing is one of the most critical strategies to monitor disease worsening^3^. Identifying worsening in VFs is difficult due to the presence of fluctuating performance, variability, and lack of a gold standard^4–7^. One approach to address this problem includes more frequent testing, though this can present a significant burden for patients while still requiring multiple years to identify progression^8–12^.

Various objective methods have been developed to help determine VF progression; these can be broadly divided into the event- and trend-based methods. Event-based methods identify progression by scoring VFs with various rules based on density and depth of defect compared to the baseline VF and have been used in major clinical trials such as EMGT, CIGTS, and AGIS^13–15^. Guided progression analysis (GPA), which is similar to the EMGT criteria, is commonly used in clinical practice and previous studies have found it identified progression sooner but with less specificity^16,17^. Trend-based methods use linear regression which can be applied to global VF parameters or pointwise data. Previous work has suggested event-based methods could identify progression sooner than trend-based methods^18,19^. Two studies compared all of these methods on a large set of longitudinal VFs and showed weak agreement, suggesting a need for a consensus among the distinct algorithms to identify progression^20,21^.

The use of artificial intelligence represents one potential approach to identify worsening earlier and more consistently^22–26^. It has even been used to predict future VF or identify patients at the highest risk of worsening^27,28^. Traditional machine learning approaches utilize pre-specified transformation of subcomponents of the data while deep learning approaches allow training models with raw data^29^. Deep learning has a variety of approaches that can be useful depending on the structure of the data. In a recent paper a specific kind of deep learning model (DLM), a convolutional long short-term memory (LSTM) model, showed success in identifying VF worsening^30^. This model is unique in that allows the extraction of spatiotemporal features which are both critical for assessing VFs.

The goal of the current work was to assess the performance of a convolutional LSTM at detecting VF worsening when trained on a consensus of event and trend-based algorithms commonly used to detect worsening. To further evaluate the robustness of the DLM we assessed its performance at identifying worsening when trained with fewer VFs. We also compare agreement among the various algorithms used to detect worsening to emphasize the importance of the need for a consensus measure of worsening. Based on the data presented here a DLM could help clinicians identify VF worsening.

## Results

**Figure 1:**
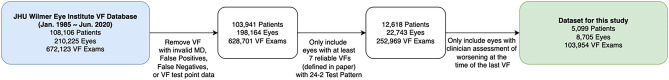
Study inclusion criteria. The flow chart shows the total number of patients, eyes, and VF exams that were present at baseline. Eyes were excluded if they did not have complete VF data and did not have at least 7 reliable fields. The final criterion for inclusion was the clinicians’ decision of worsening at the time of VF testing, which was retrospective.

8705 eyes from 5099 patients were included (Fig. 1). The median age across all patients at their first VF was 62.3 years with 56.2% female (Table 1). The initial VF mean deviation (MD) across all eyes was − 2.5 dB with a mean longitudinal decline of 0.19 dB/year. The distribution of baseline MD is shown in the histogram (Supplementary Fig. 1) Each eye had about 12 visual fields (VF) done approximately once per year. The patients were divided into training (80%), validation (10%), and test sets (10%). Table 1 displays these and additional characteristics for training, validation, and test eyes. There was no statistically significant difference among the three groups (p > 0.05, ANOVA). Using only one eye from each patient in the test set (n = 510) did not change the results (data not shown).

**Table 1:** Demographic and visual field characteristics of eyes included in this study.

	Training	Validation	Test
# Eyes (# Pts)	6.960 (4.079)	872 (510)	873 (510)
# VF/eye (SD)	11.90 (4.59)	11.95(4.75)	12.07(4.78)
Avg. time between VFs in year (SD)	1.04 (0.37)	1.05 (0.35)	1.07 (0.41)
Median age at first VF in year (SD)	62.18 (12.03)	63.64 (11.52)	62.42 (12.00)
Avg. initial false positives in % (SD)	0.05 (0.68)	0.07 (0.80)	0.03 (0.50)
Avg. initial false negatives in % (SD)	2.05 (5.42)	1.66 (5.17)	2.14 (5.52)
Avg. initial fixation losses in (SD)	0.13 (0.19)	0.13 (0.18)	0.13 (0.17)
Female in % (# Pts)	55.4% (2.258)	58.8% (300)	60.6% (309)
Ethnicity in % (# Pts)			
White	62.8% (2.561)	61.2% (312)	60.0% (306)
Black	25.7% (1.048)	26.1% (133)	28.8% (147)
Asian	4.3% (175)	4.7% (24)	4.7% (24)
Other	3.0% (124)	2.9% (15)	3.5% (18)
Unknown	4.2% (171)	5.1% (26)	2.9% (15)
Avg. initial MD in dB (SD)	− 2.51 (3.86)	− 2.19 (3.76)	− 2.69 (4.04)
Avg. MD slope in dB/year (SD)	− 0.17 (0.42)	− 0.19 (0.38)	− 0.17 (0.43)

**Figure 2:**
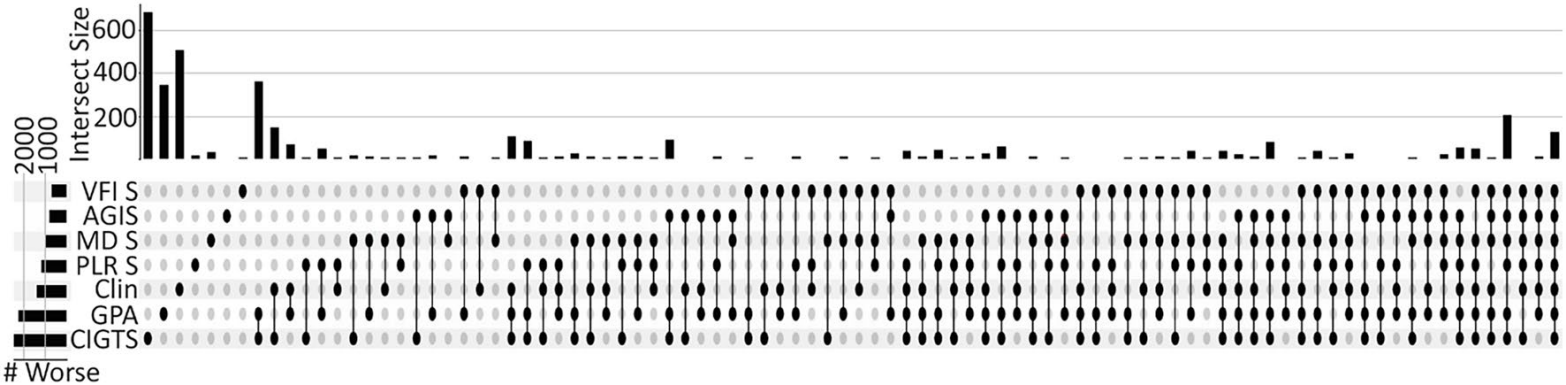
Upset plot with all methods to detect worsening. Each row in the table corresponds to a different method to detect worsening. The bar chart on the left indicates the total number of eyes identified as worsening by the indicated method with the gray lines identifying 1000 and 2000. The columns indicate, with dots and lines, the combination of methods being assessed. The bar chart above the column shows the number of eyes progressing for that specific combination of methods. The first seven columns show how many were identified as progressing by each method alone while the rightmost column shows how many eyes were identified as progressing by every method.

For each eye, all methods to assess progression were computed and the results are shown in Fig. 2. This plot shows the total number of progressing eyes on the left of the rows near each method; CIGTS had the highest number of progressing eyes with 2411 (27.7%) followed by GPA with 2192 (25.2%). VFI slope and AGIS identified the fewest progressing eyes with 643 (7.4%) and 784 (9.0%), respectively. The clinicians were in the middle identifying 1353 (15.6%) progressing eyes. The columns show the number of eyes that had progression based on various combinations of methods in each row, in total 126 eyes were found to be progressing by all methods and clinicians (rightmost column).

Kappa coefficients to compare the agreement among each method are shown in Table 2. The agreement across all methods of detecting VF worsening was calculated and Fleiss kappa (95% CI) was 0.34 (0.33, 0.36) when clinicians’ assessments of worsening were included and 0.41 (0.39, 0.42) when the clinician assessments were not included. Trend-based methods (MD slope, PLR slope, and VFI slope) in general had a higher agreement among themselves (darker shade). Of the event-based methods (AGIS, GPA, and CIGTS) CIGTS had the least agreement with other trend and event-based methods. The clinicians’ assessment of worsening had a weak agreement with all other methods.

**Table 2:**
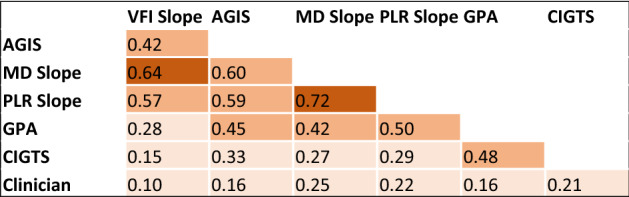
Kappa coefficients to assess agreement across methods to define VF worsening.

The level of agreement is highlighted by the color in the table with a darker shade indicating greater agreement.

**Figure 3:**
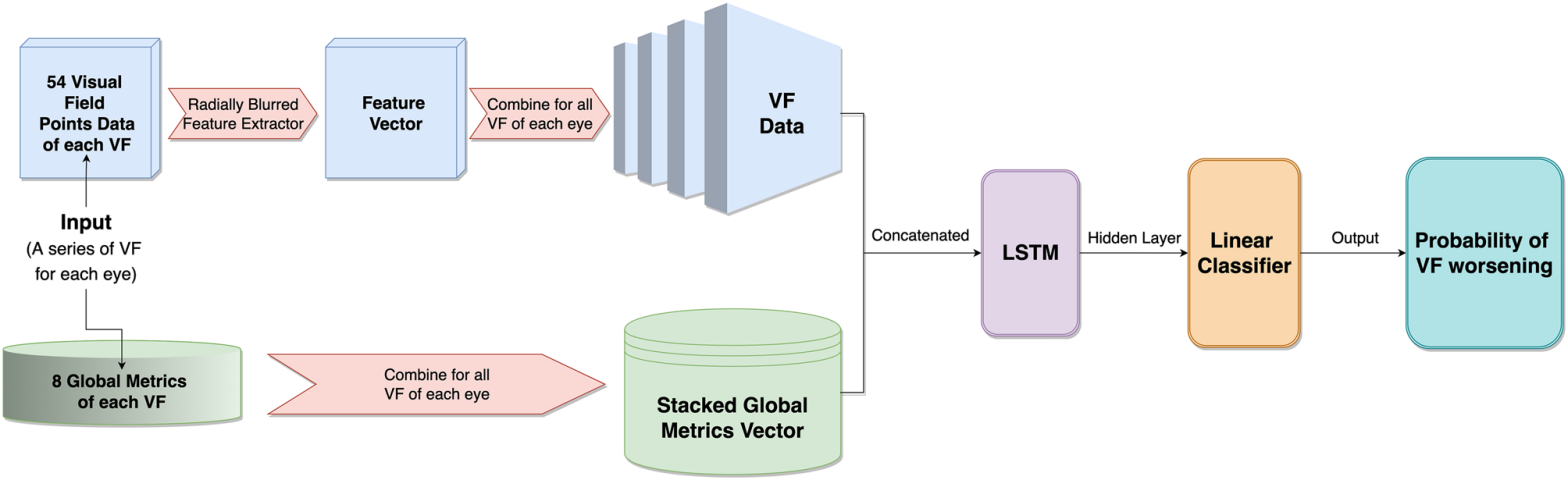
Deep learning model diagram. Deep learning architecture that incorporates data from visual fields and their 8 global metrics.

**Figure 4:**
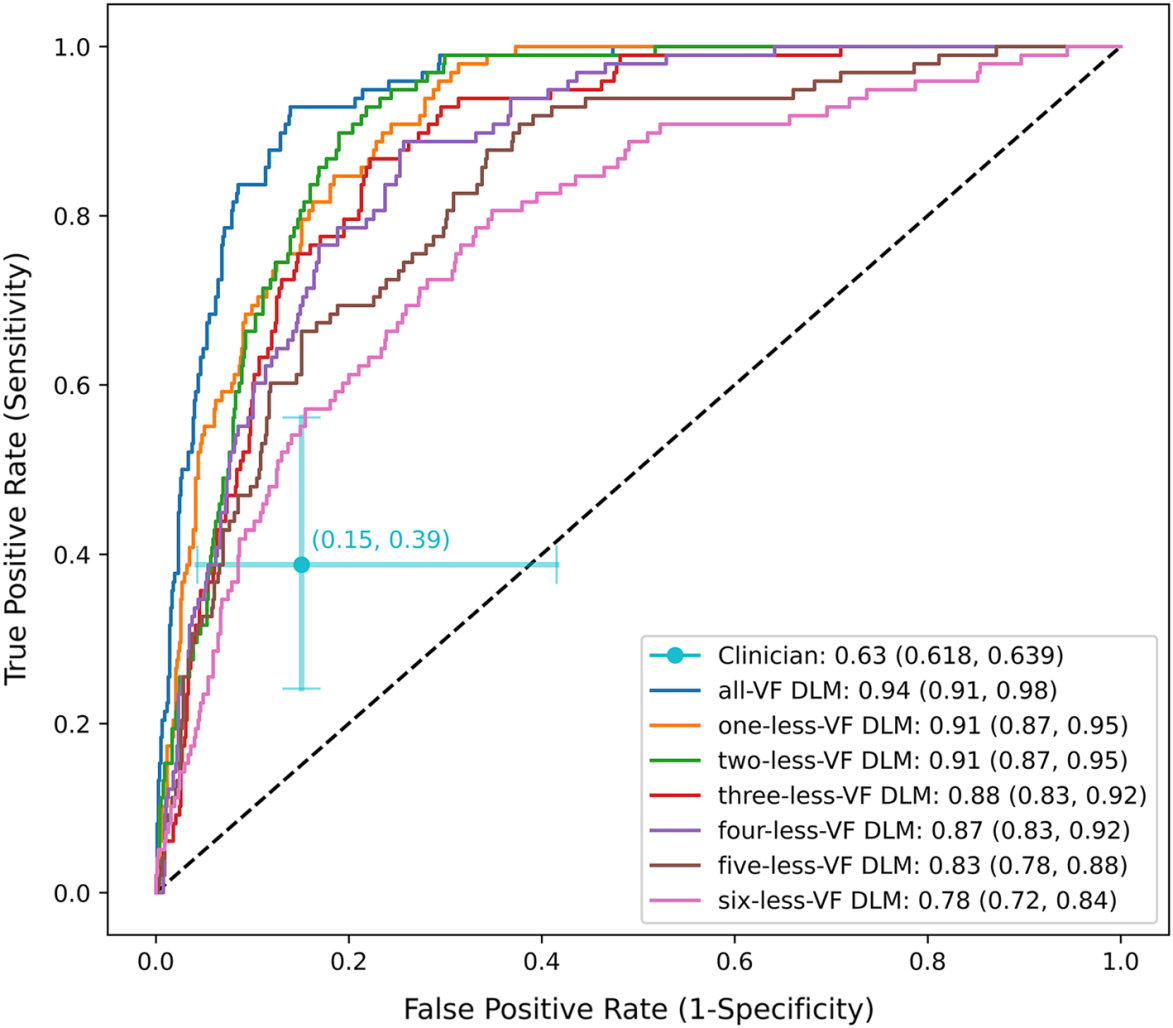
Test set performance of deep learning model and clinician assessment of VF worsening. The blue line shows the model performance with the full data. A decreasing AUC can be seen with the removal of more VFs (rightward shift of ROC curve) to the pink, where 6 of the last VFs were removed. The AUC decreased from 0.94 (0.91, 0.98) to 0.78 (0.72, 0.84) when comparing the full data and removal of 6 VFs, respectively. The cyan dot and 95% CI whiskers show the sensitivity and specificity of clinicians in detecting worsening in the same set of eyes during routine clinical practice. The estimated AUC for clinicians was 0.63 (0.62, 0.64).

The deep learning model (DLM) was trained to detect the worsening of visual fields based on the 4 of 6 reference standard (Fig. 3). The DLM had an AUC (95% CI) of 0.94 (0.93, 0.99) (blue line, Fig. 4). In the ROC plot (Fig. 3), the clinician assessment of worsening is demonstrated to have a lower true positive rate (TPR) and higher false positive rate (FPR) than the DLM. The clinician assessment had a TPR (95% CI) of 0.42 (0.32, 0.54) and an FPR (95% CI) of 0.16 (0.06, 0.37). At the clinician’s TPR (0.42), the all-VF DLM had an FPR (95% CI) of 0.024 (0.00, 0.062). At the clinician’s FPR (0.16), the all-VF DLM had a TPR of 0.93 (0.87, 0.99). The estimated AUC for clinicians was 0.63 (0.62, 0.64). One benefit to applying a DLM is that the model performance can be assessed with fewer data points. For each eye, up to the most recent 6 VFs were removed and the model performance was assessed (multi-colored lines). The AUC decreased with the removal of more VFs but all AUCs were still significantly larger than the clinician assessment using all the VF Data (p < 0.001 for all models compared to the clinician). The DLM had a significantly higher AUC regardless of how many tests (1 out of 6 to 6 out of 6) were required for the reference standard (Supplementary Table 1). The mixed-effects model also had a lower AUC than the DLM with an AUC of 0.82 (0.77–0.86, data not shown).

Table 3 shows sensitivity and specificity for the DLM and clinicians after subdividing the data based on initial disease severity. The performance is significantly worse for both the DLM and clinicians when patients had more severe disease at baseline (p < 0.05 for both comparisons).

**Table 3:** Performance of deep learning model and clinicians at identifying VF worsening in a specific subpopulation of patients.

	Clinician estimated AUC (lower bound, upper bound)	Deep learning AUC (95% CI)
Initial MD in dB < − 6	0.50059 (0.50055, 0.50063)	0.85 (0.73, 0.97)
Initial MD in dB ≥ − 6	0.67 (0.66, 0.68)	0.96 (0.92, 0.99)

A similar analysis was conducted using the clinician assessment of worsening as the reference standard and the DLM was also able to successfully identify worsening with an AUC of 0.79 (Supplementary Fig. 2). The comparison of AUC for disease severity is also shown (Supplementary Table 2).

## Discussion

In this large population of patients, there was significant variability in agreement among the various methods to identify VF worsening. We show that a DLM trained to identify VF worsening based on a consensus of these methods performed well. Additionally, the DLM was robust and had a significantly higher AUC than clinician performance and the mixed-effects model when provided with less VF data than available to the clinician. Both the DLM and clinicians had more difficulty assessing worsening when the disease at onset was more advanced. The DLM can help clinicians better assess when VF is worsening.

Multiple studies have compared agreement among algorithms for identifying VF worsening. Initial studies showed event-based methods, namely GPA, had more sensitivity and earlier detection of worsening compared to trend-based, namely VFI and MD regression^18,19^. Various studies comparing event and trend-based methods show variation in agreement ranging from poor to moderate with kappa coefficients ranging from 0.22 to 0.51^18–21^. Agreement within event-based methods is better, ranging from 0.48 to 0.55. Trend-based methods also have high agreement up to 0.67 between MD and VFI, but also as low as 0.2 between MD and PLR^20,21^. Our study also showed moderate agreement between GPA and both AGIS (0.45) and CIGTS (0.48). We found higher agreement among distinct trend-based methods, ranging from 0.57 to 0.72. One unique strength of our study was assessing GPA agreement in a large sample. The other study with a large sample (~ 13,000 eyes) did not assess GPA^20^. Interestingly, the percentage of eyes identified as worsening has varied across studies. Our results contrast with a recent report that found PLR had the highest proportion of VFs progressing at almost 50% and CIGTS was the lowest at 10%^20^. Another report found CIGTS/GPA/PLR identified worsening in the highest number of eyes, while VFI rate was the lowest which is more similar to our findings^21^. Importantly, the differences here could arise from variability in patient population and practice patterns. The eyes in this study had more mild disease at baseline with mean MD of − 2.5 dB compared to about − 5 dB in the other studies. The demographics of patients in this study are comparable to other studies though there is a higher percentage of female and Black patients than seen in population studies^31^.

A range of factors may underlie when algorithms agree. With more VF tests trend-based methods were able to find progression compared with GPA^18^. To specifically assess discordance one study identified eyes where 3/6 algorithms identified worsening and the other 3 showed no worsening and found that discordance was associated with worse initial MD, older age, more VFs, longer follow-up duration, and institution from which data was from^20^. These findings highlight the difficulty in identifying any single method as an objective reference standard. Even the decision of clinical experts shows significant variation^32,33^. In this study, we combined objective metrics to identify a consensus. Requiring consensus of too many algorithms would create too much stringency; for example, 5/6 and 6/6 algorithms agreement in one study found worsening in only 3.1% and 2.5%, respectively^20^. In this study, the percentage of patients identified as worsening with 4, 5, and 6 algorithms identifying worsening was 10.0%, 6.8%, and 3.8%. We applied the definition of consensus as 4/6 or more agreement. One strength of requiring four algorithms was that any eye identified as worsening required at least one event- and trend-based method to agree. Though our major focus here uses the consensus decision as a reference standard we also conducted a supplementary analysis using the clinician decision as the reference standard. The DLM was successfully trained with an AUC of 0.79. This worse performance, when compared with the consensus as the reference standard, could be due to numerous reasons such as less algorithmic approach by the clinicians or the inclusion of clinical factors which are not available to the model.

Traditional machine learning has been applied to glaucoma for many years and more recent advances in computing have allowed more complex models^29^. Since VF changes have a significant spatiotemporal component, a recent paper showed success using a convolutional LSTM (cLSTM) model which retains spatial and temporal features. In that study, the changes in VF were defined by trend-based methods and it was shown that cLSTM identified worsening successfully with AUC values as high as 0.939^30^. These values are higher than what was seen in traditional machine learning approaches, for example, the Gaussian mixture model had sensitivity and specificity of 89.9% and 93.8% with an AUC of 0.86^22^. However, these studies are all difficult to compare given the various reference standards. This study is unique in that cLSTM is used to identify VF worsening based on the consensus of multiple algorithms. We also compare the DLM with a mixed-effects model and show superior performance. Another comparison in this study is clinician performance which demonstrates the potential value of the DLM in routine clinical care. Though the clinician performance here has limitations, to our knowledge, this is the first study to show clinician performance in a large dataset and compare it to a DLM^34^. Previous deep learning studies have shown excellent results such as excellent accuracy^30^, ability to predict future VFs^27,28^, and earlier identification of progression^35^. However, comparison of deep learning performance with clinicians will be critical if such models will be deployed in a clinical setting to assess for worsening. Since other studies had shown the successful ability of deep learning to forecast future VFs, we assessed the performance of the model after removing the final VFs. Removing each additional VF caused worse performance of the model, but even after removing 5 of the most recent VFs the DLM performs as well as a mixed-effects model. These findings show that deep learning is not only for accurate identification of disease diagnosis or detection of progression, but also may identify early markers for higher-risk patients.

This study has some limitations. The data is retrospective and from a tertiary referral center. Additionally, there was some filtering of the data to include only those eyes with longitudinal data and reliable VFs to allow accurate identification of worsening. This could create bias in selected patients and limit the results' generalizability. Though it is important to note the patients throughout the disease severity spectrum were included in the study. External validation of our cLSTM mode will be required before this model can be deployed for clinical use. The VF data in this study was based on SITA 24-2 testing from Zeiss Humphrey Field Analyzer, utilization of other VF data (e.g. Haag-Streit Octopus Perimeter) would require representative training data of other tests. Another limitation is that the clinician assessment for worsening was made retrospectively and at a single time point at the last visual field and the clinicians were not specifically instructed in how to grade this assessment. However, the clinicians represent glaucoma specialists during routine clinical care who had access to all visual fields as well as progression diagrams containing GPA and MD/VFI slopes. Some future directions include further comparisons into deep learning and clinician performance in more controlled and prospective settings as well as the role of including additional parameters such as clinical data or structural testing in the assessment of worsening.

In conclusion, we show that there is significant variability among the objective methods to classify VF worsening and that the consensus of these methods represents one method to create a reference standard. Using this reference standard, we show that a DLM, specifically cLSTM, can successfully identify VF worsening and would help support clinicians during routine clinical care. After careful external validation, such models may be deployed to identify VF worsening accurately and automatically in glaucoma clinics.

## Methods

### Consent waiver

This study was reviewed and approved by the Johns Hopkins University School of Medicine Institutional Review Board and adhered to the tenets of the Declaration of Helsinki. The requirement for informed consent was waived because of the retrospective nature of the study.

### Data collection

Demographic and clinical data were obtained from patients seen at the Johns Hopkins Wilmer Eye Institute from June 1990 to June 2020. The clinical assessment of worsening at the last visual field (VF) was extracted from Epic (Verona, Wisconsin). Clinicians rating eyes either possibly or likely worsening on VF testing were labeled as worsening, while other choices (stable, possibly, or likely improving) were labeled as not worsening. The VF data were HVF 24-2 studies extracted from FORUM (Zeiss, Dublin, CA). The majority of these were SITA-Standard but it also included SITA-Fast, full threshold, and SITA-Faster.

VFs were included only if they were considered reliable with less than 15% false positives and less than either 25% false negative for mild/moderate disease or 50% for severe disease^36^. We only included eyes with at least 7 reliable VFs so that an accurate determination of longitudinal change could be made. The last VF in the series for each eye was required to have a clinician assessment of VF worsening or not worsening recorded in the charts. The number of VF tests excluded at each step is shown in the flow chart (Fig. 1).

### Methods to determine visual field worsening

There is no gold standard to assess VF worsening but there are numerous algorithms that have been commonly employed in the field. We used six of these automated methods. This includes three event-based methods: Guided Progression Analysis (GPA), Advanced Glaucoma Intervention Study (AGIS) scoring system, and Collaborative Initial Glaucoma Treatment Study (CIGTS) scoring system. We also used three trend-based methods: Mean deviation (MD) rate of change (MD slope), VF index (VFI) rate of change (VFI slope), and Pointwise linear regression (PLR). In addition to these algorithms, we also had access to clinician assessment of worsening for the last VF in each series. The description of each of these methods is outlined below. In all event-based methods, a baseline was needed which was calculated as the average of the first two VFs.

GPA is typically calculated by proprietary software and based on the Glaucoma Change Probability Analysis ^3,21,37^. Deviation values at each point in the VF are compared to the average of the values at the first two VFs. The points with a difference significantly higher than the test–retest variability at a p < 0.05 are identified. As we did not have access to the GPA database for thresholds for test–retest variability we determined thresholds for α < 0.05 based on an empiric normative database from the University of Iowa. We also used total deviation values instead of pattern deviation which is classically used by GPA, as previous studies have shown total deviation is more likely to detect progression^38^. We defined worsening as any three or more points worsening beyond the threshold level for three consecutive fields compared to the average of the first two VF exams.

AGIS score was calculated for each VF as described in the AGIS trial^13^. Briefly, each VF is graded based on the depth and number of defects in pre-specified locations on the VF. These pre-specified locations include nasal, superior, and inferior hemifields. The score ranges from 0 to 20 and scores for each VF are compared to the baseline scores. A computer program was used to calculate the score^39^. An AGIS score increase of at least four points which is sustained in three consecutive VFs was classified as worsening.

CIGTS score calculation has been previously described in the CIGTS trial^15^. This score uses the total deviation probability map and is calculated based on the density and depth of defects across the VF. VFs with multiple isolated points with defects would receive a lower score than when there were clusters of points with defects. The CIGTS score also ranges from 0 to 20 and an increase of three or more test points which is sustained for three consecutive VF was classified as worsening.

The MD slope was calculated as the simple linear regression of the MD values for the VFs. VF worsening was defined as a negative slope ≤ − 0.5 dB/year with a regression p-value less than 0.05. Similarly, the VFI slope was calculated as the linear regression of the VFI values. VF worsening was defined as a negative slope ≤ − 1.8%/year with a p-value of less than 0.05^21^.

For PLR, linear regression was performed for the total deviation values of each of the 52 VF points separately. VF worsening was defined as the presence of any three points with a negative slope ≤ − 1 dB/year with a p-value ≤ 0.01^21^.

Clinician assessment of worsening was determined for each eye by the clinician at the time of the last visual field and recorded in Epic. The clinician could choose from checkboxes that denoted likely worsening, possible worsening, stable, possible improvement, or likely improvement. A decision of likely or possible progression was classified as worsening while all other choices were classified as not worsening.

### Reference standard

A reference standard for VF worsening was defined as at least four out of six algorithms (GPA, AGIS, CIGTS, MD slope, VFI slope, and PLR) identifying worsening. This was used as the label for worsening to train/test the deep learning model (DLM) and serves as the ground truth for VF worsening in this study. This reference was also used as the reference for the receiver-operating characteristic (ROC) curve in Fig. 4. A supplementary analysis was conducted with the clinician assessment of worsening for worsening used as the reference standard for training the DLM and generating the ROC curve (Supplementary Fig. 2).

### Deep learning architecture

The DLM architecture is described in Fig. 1. The input to the network consists of two parts: (1) a set of 7 or more VF images, each image has 54 points which were radially blurred onto a 12 × 12 grid and stacked together; (2) a stack of 7 or more sets of 8 global metrics from each VF (Age, VFI in %, PSD in dB, MD in dB, False Negatives in %, False Positives in %, Test Duration in sec, and Fixation Losses). The DLM architecture can receive unevenly spaced temporal data from each VF series. The dataset was split into 80%, 10%, and 10% for training, validation, and testing, respectively. The data was split on a patient level so if both eyes were included, they would fall within the same set. Including only one eye from each patient did not change the results of the study. The data were randomly distributed so all datasets, training, validation, and testing consisted of eyes that were and were not determined to be worsening. For the deep learning architecture, we implemented a single 2D convolutional LSTM with a 3 × 3 kernel size. Batch normalization was also integrated into the model to reduce internal covariate shift. The output of the model was the probability of VF worsening.

An additional analysis was carried out by removing VFs from the end of the series of VFs that were included for each eye and re-training the model with fewer data points. This tested the DLM’s ability to judge worsening before it had access to all of the information used by the 4 out 6 algorithms reference standard. The VFs were removed sequentially from the end (removing the final VF, removing the final two VFs, removing the final three VFs, etc.). This was done up to a maximum of removing the final 6 VFs since all included eyes required at least 7 VFs. This allowed each eye to have at least 1 VF entered the model as input, though about 87% of eyes had more than this minimum number. The label for worsening and assessment of performance was still based on the original consensus of 4 out of 6 using all the VFs.

### Statistical analysis

Since multiple methods were used to identify VF worsening, we wanted to calculate the level of agreement among these methods. The pairwise agreement was identified based on Cohen’s kappa coefficient. Based on previous literature a kappa coefficient of 0 to 0.2 indicated slight agreement, 0.2 to 0.4 fair agreement, 0.4 to 0.6 moderate agreement, and 0.6 to 0.8 substantial agreement^40^. Agreement across more than two methods was also determined by calculating the Fleiss’ kappa coefficient^41^.

Another model for identifying worsening was created using a mixed-effects model that was provided with all the same data as the LSTM (Fig. 3) with “Patient ID” and “Eye ID” treated as random effects and all other features treated as fixed effects.

For the deep learning prediction, we constructed a ROC curve, which can visualize the performance of the DLM at all classification thresholds (Fig. 4). An AUC value and its 95% confidence interval were calculated as a measure of prediction performance. The Clopper-Pearson method was used to calculate the 95% confidence interval of false positive rates and true positive rates^42^. The same approach was used to identify an AUC for the mixed-effects model approach. For clinician assessment of worsening a fixed true positive rate and false positive rate was calculated. An exact ROC curve cannot be calculated for clinician assessment of worsening since it is a discrete and binary classification. To evaluate clinician prediction performance, a best minmax AUC score and its upper and lower bounds were calculated, assuming the clinician ROC curve is concave or monotone^43^.

Unless specified otherwise all comparisons and performance analyses were calculated on the test dataset only. The DLM was developed using Python (Python Software Foundation, Wilmington, Delaware). SPSS was used for statistical comparisons (IBM Corp, Armonk, NY).

### Conference presentations

American Glaucoma Society, Paper Presentation, Nashville, TN, 2022.

## Supplementary Information

Supplementary Information.

## Data Availability

The datasets generated and/or analyzed during the current study are not publicly available due to being protected health information. The raw data would not be available to share.
