# Supplementary material for: A deep learning model incorporating spatial and temporal information successfully detects visual field worsening using a consensus based approach
Source: Sci Rep. 2023 Jan 19;13:1041. doi: 10.1038/s41598-023-28003-6 (PMC9852268; doi:10.1038/s41598-023-28003-6)
Supplement: Supplementary file 1 — Supplementary Information. [file 41598_2023_28003_MOESM1_ESM.docx]

**Title Page**

1. **Title (150 characters):** A Comparison of Clinician and Deep Learning Performance at Detecting Visual Field Worsening
2. **Running head (60 characters):** Comparing Clinician and Deep Learning at Detecting VF Worsening
3. **Authors:** Jasdeep Sabharwal, MD, PhD^1^*, Kaihua Hou^2^*, Patrick Herbert^2^, Chris Bradley, PhD^1^, Chris A. Johnson, PhD^3^, Michael Wall, MD ^3^, Pradeep Y. Ramulu, MD, PhD^1^, Mathias Unberath, PhD^2,^ Jithin Yohannan, MD, MPH^1,2, +^

^1^Wilmer Eye Institute, Johns Hopkins University School of Medicine, Baltimore, Maryland.

^2^Malone Center for Engineering, Johns Hopkins University, Baltimore, Maryland

^3^Department of Ophthalmology and Visual Sciences, University of Iowa, Iowa City, IA, USA.

*These authors contributed equally

+Corresponding author

**Supplemental Figures**

**
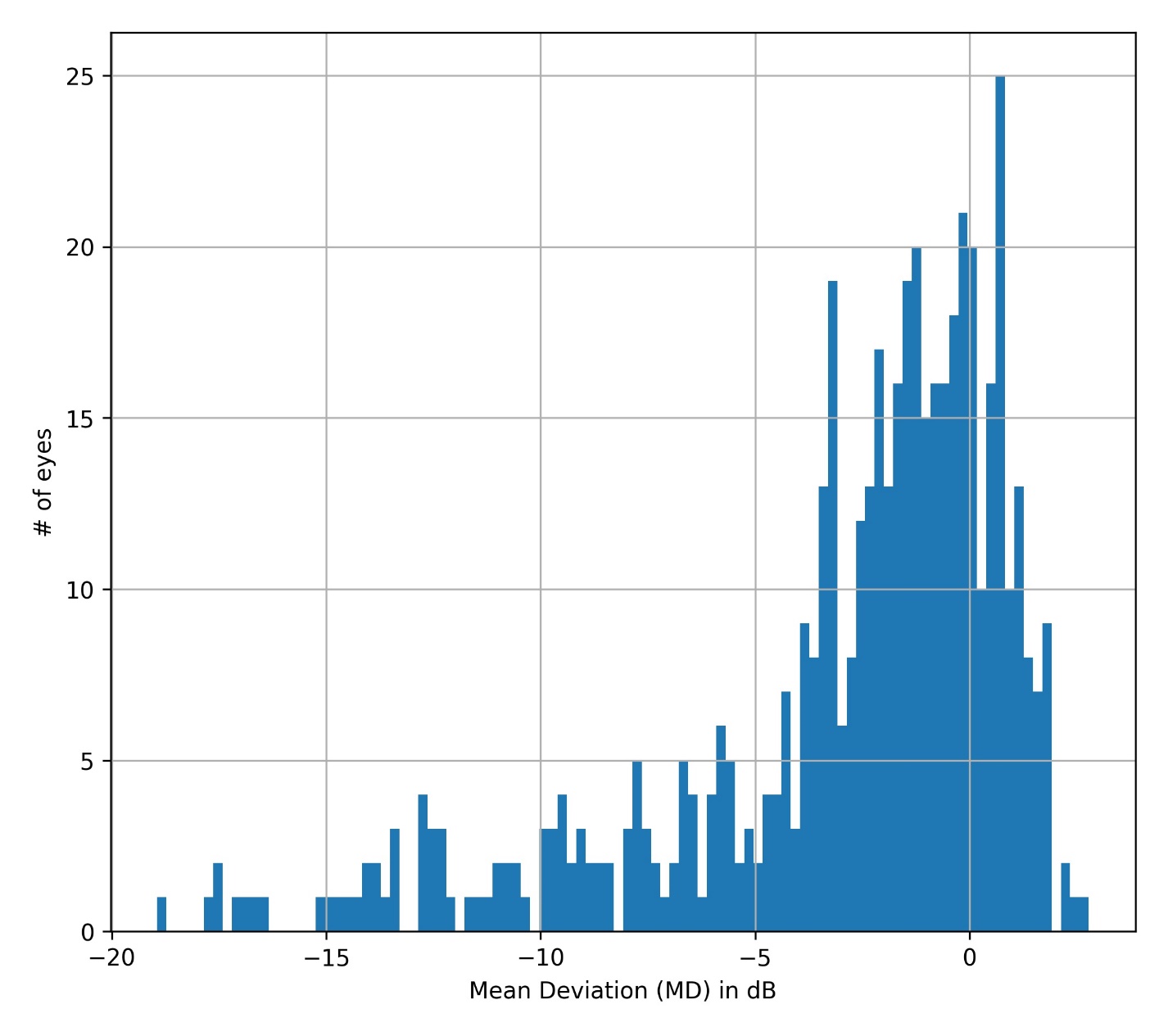
**

**Supplementary Figure 1 –** **Distribution of initial mean deviation (MD) across the test set.** The histogram shows the distribution of MD across all patients in the test set. The mean and SD were -2.69 and 4.04 dB.


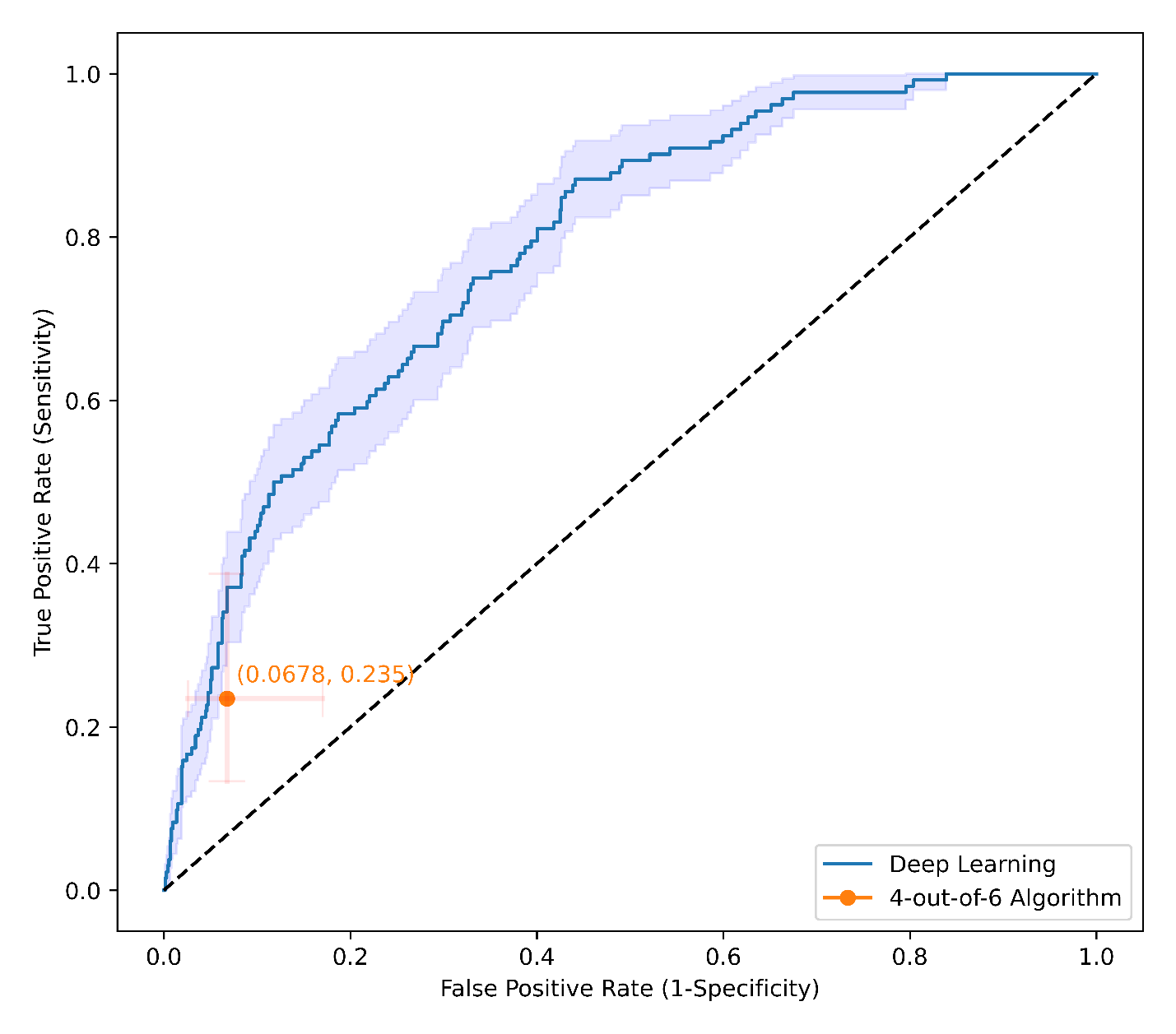


**Supplementary Figure 2 –** **Test set performance of deep learning AI and classic algorithms.** Reference standard for VF worsening based on clinician assessment of worsening. The ROC curve demonstrates the performance of the deep learning model trained to identify VF worsening (blue line) with its 95% CI (shaded blue region). The AUC (95% CI) for the DLM was 0.79 (0.74, 0.83). The orange dot and 95% CI whiskers show sensitivity and specificity of agreement between automated methods. The estimated AUC for the agreement between automated methods was 0.59 (0.58, 0.59)

| **Reference Standard**  **(Frequency)** | **All-VF DLM**  **AUC (95% CI)** | **Clinician**  **AUC (95% CI)** |
| --- | --- | --- |
| **1 out of 6**  **(3206/8705)** | 0.86 (0.83, 0.89) | 0.59 (0.587, 0.596) |
| **2 out of 6**  **(1899/8705)** | 0.91 (0.88, 0.94) | 0.62 (0.61, 0.62) |
| **3 out of 6**  **(1257/8705)** | 0.94 (0.91, 0.97) | 0.64 (0.63, 0.64) |
| **4 out of 6**  **(869/8705)** | 0.94 (0.90, 0.99) | 0.64(0.63, 0.66) |
| **5 out of 6**  **(591/8705)** | 0.97 (0.94, 1.00) | 0.65 (0.64, 0.66) |
| **6 out of 6**  **(330/8705)** | 0.98 (0.94, 1.00) | 0.63 (0.62, 0.64) |

**Supplementary Table 1 – Comparing model and clinician performance with variable reference standards.** The leftmost column shows varying levels of stringency for the reference standard from only needing one algorithm to identify worsening (top row) to requiring all six (bottom row). The AUC is shown for the DLM and clinician. All show significantly higher AUC for the DLM.

|  | **4-out-of 6 Algorithm Estimated AUC***  **(Lower bound, Upper bound)** | **Deep Learning AUC***  **(95% CI)** |
| --- | --- | --- |
| **Initial MD in dB < -6** | 0.50  (0.50055, 0.50063) | 0.732  (0.586, 0.879) |
| **Initial MD in dB >= -6** | 0.599  (0.596, 0.602) | 0.804  (0.754, 0.853) |

**Supplementary Table 2 – Deep learning and classic algorithm performance divided by patient factors**. *See methods.
